# Unclosed wound: effect of childhood access to healthcare on cardiovascular health trajectories of Chinese older adults based on entropy balancing analysis

**DOI:** 10.3389/fpubh.2025.1602953

**Published:** 2025-07-02

**Authors:** Boshu Mao, Xiaoyi Fang, Lingjun Liu

**Affiliations:** ^1^School of Social Development and Public Policy, Fudan University, Shanghai, China; ^2^Institute of Population Research, Peking University, Beijing, China

**Keywords:** childhood access to healthcare, cardiovascular health, group-based trajectory modeling, entropy balancing, older adults

## Abstract

**Background:**

Cardiovascular Diseases (CVD) remain a leading threat among aging populations globally, with Cardiovascular Health (CVH) trajectories shaped by cumulative exposures across the life course. Understanding these life-course connections is urgent to inform equitable geriatric care strategies.

**Objective:**

This study aims to examine the long-term trajectories of CVH and the association between Childhood Access to Healthcare (CAH) and CVH trajectories in Chinese older adults.

**Methods:**

Data were obtained from Chinese Longitudinal Healthy Longevity Study (CLHLS). A composite CVH score was established based on the American Heart Association’s (AHA) guidelines. Group-Based Trajectory Modeling (GBTM) was employed to identify distinct CVH trajectories over time. Multi-logistic regression was used to analyze the association between CAH and CVH trajectories. To minimize potential confounding and selection bias, entropy balancing was applied to balance covariates between the treatment and control groups.

**Results:**

Three distinct CVH trajectories were identified: Low-rapid decline (25.2%), Moderate-stable (65.7%), and High-stable (9.1%). Compared with high-stable trajectory, individuals with CAH were associated with lower likelihood in moderate-stable trajectory (Adjusted and balanced OR = 0.61, 95% CI: 0.44–0.85, *p* < 0.01) and low-rapid decline trajectory (Adjusted and balanced OR = 0.63, 95% CI: 0.44–0.90, *p* < 0.05), suggesting that CAH was associated with more favorable long-term CVH outcomes. Subgroup analysis indicated that the association was generally stable across different populations.

**Conclusion:**

CAH significantly influences the long-term CVH trajectories of older adults in China. These findings underscore the need for public health interventions that prioritize childhood healthcare access to reduce the burden of CVD in the aging population.

## 1 Introduction

Population aging is a global trend, with estimates suggesting that by 2050, one in six individuals worldwide will be aged 65 years or older (1). The aging population poses a significant challenge to global public health. Cardiovascular health (CVH) is one of the key aspects. in China, It is estimated that approximately 330 million individuals are affected by Cardiovascular Disease (CVD) (2). Assessing CVH and conducting research on early pathological changes to enhance prevention, treatment, and understanding of CVD have become important areas of consensus in public health. In CVH assessment, the American Heart Association (AHA) introduced a composite marker of CVH in 2010, which consists of four behavioral and three biological metrics (3). These CVH metrics are excellent predictors of CVD, mortality, and many essential health outcomes across various populations (4, 5). The value of the total CVH metrics surpasses that of any of its individual components (6). Various types of CVD increase with age, and the outcomes tend to be more detrimental (7). CVH metrics are generally poorer in older adults, who face higher risks of CVD (8). For China, the number of older adult’s individuals is expected to reach 418 million by 2035 (9). This increase in the older adult population makes CVH a greater public health concern. Recently, some studies have examined long-term patterns in CVH metrics, showing that over time, these metrics tend to follow different trajectories (10–12). However, research on CVH metric trajectories in older adults remains limited, especially in China, where most studies have focused solely on the cross-sectional CVH status of the older adults (13, 14). Long-term patterns of CVH in older Chinese adults still require further investigation (15).

Life course theory provides an important perspective in studying aging and health. According to life course theory, experiencing adverse or positive living conditions or events during this period can influence later health outcomes including CVH (16). Healthcare is one of the major influencing factors of health throughout the life cycle (17). Adequate healthcare services during childhood are essential for ensuring health levels, which in turn affect health status in later stages of life through cumulative effects (18). During the early years of the People’s Republic of China, the country faced multiple burdens on healthcare resources, including infectious diseases and maternal, child, and infant health issues. Many children during this period were unable to access sufficient healthcare services (19). The various disadvantages experienced in early life tend to accumulate over time, and the aging process of individuals is largely shaped by the advantages and disadvantages encountered during their formative years (16). Much of the existing research has focused on the influence of childhood family environments on later-life health outcomes (20, 21), while neglecting to examine whether older individuals had their medical needs adequately met during childhood (17, 22). Furthermore, the findings of these studies are predominantly limited to midlife outcomes (22). The long-term impact of Childhood Access to Healthcare (CAH) on CVH in later life remains underexplored in the literature. Investigating the relationship between childhood healthcare and later-life CVH trajectories in Chinese older adults is crucial for developing effective public health strategies and reducing the burden of CVD (23). If childhood health exerts long-term effects on aging, then ensuring access to pediatric healthcare could yield substantial public health and economic benefits (24). This is particularly true for CVD, which impose a major global public health burden (24).

This study aimed to examine the long-term trajectories of CVH and the association between CAH and CVH trajectories in Chinese older adults. We used data from the Chinese Longitudinal Healthy Longevity Study (CLHLS) from 2008 to 2018. First, based on the AHA guidelines, we established a CVH score index. Then, we used the Group-Based Trajectory Modeling (GBTM) to analyze the long-term patterns of CVH score in Chinese older adults from 2008 to 2018. We examined the relationship between CAH and CVH score using multi-logistic regression models. To control for potential confounding factors, we also performed a sensitivity analysis with entropy balancing. Subgroup analysis has also conducted to explore the heterogeneous effect of CAH on CVH.

## 2 Materials and methods

### 2.1 Study population

The data used in this study was obtained 4 waves from 2008 to 2018 in CLHLS database. CLHLS is a longitudinal survey of the older adults organized by Peking University Center for Healthy Aging and Development, covering 23 provinces, municipalities and autonomous regions in China. The latest survey was conducted in 2018. CLHLS is the earliest and longest social science survey in China (25). In the CLHLS, a total of 2,440 participants completed all survey waves from 2008 to 2018 (26). After excluding individuals with missing data on CVH (*N* = 450), the final analytical sample comprised *N* = 1990 subjects.

### 2.2 Definition of CVH score

**Figure 1:**
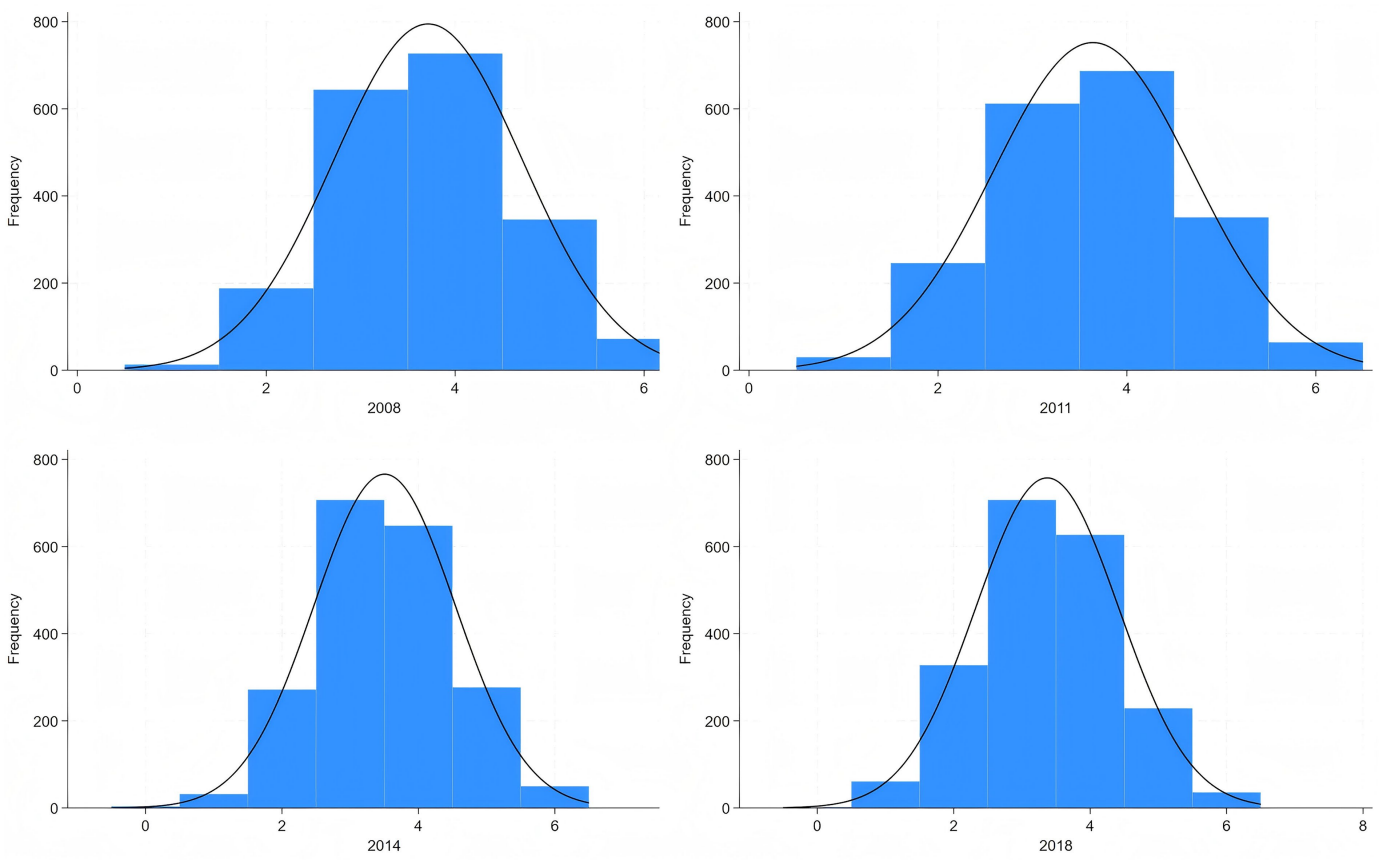
Distribution of CVH score.

Based on the AHA guidelines and existing research on CLHLS (3, 14), CVH score is composed of six dimensions, as total Cholesterol if not applicable in CLHLS: hypertension, diabetes, exercise, BMI, diet, and smoking. We constructed a CVH score ranging from 0 to 6, with higher scores being associated with more ideal CVH conditions. The specific score construction system can be found in Table 1 and the distribution of CVH score can be found in Figure 1. Although the sleep dimension has been newly incorporated into the AHA’s updated Life’s Essential 8 metrics in 2022, the optimal sleep duration for older adult remains scientifically debated when measurement methodologies lack standardization (27). Given these methodological inconsistencies in sleep assessment approaches, we excluded the sleep component from our analytical framework.

**Table 1:** Measurement of CVH score.

Dimensions	Measurement
Hypertension	Participants with self-reported or diagnosed hypertension = 0, others = 1
Diabetes	Participants with self-reported or diagnosed diabetes = 0, others = 1
Exercise	Participants who currently engage in regular exercise = 1, others = 0
BMI	Participants with a BMl between 18.5 and 24 kg/m^2^ = 1, others = 0
Diet	A modified healthy diet score based on the intake frequency of fruits, vegetables, fish, bean products, and tea. “always or almost every day” = 2. “sometimes or occasionally” = 1. “rarely or never” = 0. The scores were summed and participants with a score higher than 6 = 1, others = 0.
Smoking	Participants who currently smoke = 0, others = 1.

### 2.3 Definition of CAH

Following the approach used in existing studies (28). We used self-perceived childhood healthcare to measure the CAH of the older adults (29). In the CLHLS, respondents were asked about their childhood healthcare condition using question F63: “Could you get adequate medical service when you were sick in childhood? “, if the answer was “Yes,” it was defined as Accessible to healthcare in childhood and assigned a value of 1; otherwise, it was assigned a value of 0. Older adults’ retrospective recall of CAH effectively captures multidimensional healthcare circumstances, providing a more comprehensive assessment than single objective indicators (29). As a critical component of childhood socioeconomic status, this recall-based measure of CAH has demonstrated criterion validity through significant associations with multiple geriatric health endpoints (30, 31).

The self-reported measure of CAH may be subject to recall bias. However, the CLHLS study design incorporated repeated measurements by readministering the same CAH question to participants across three survey waves (2008, 2011, and 2014). To further address potential recall bias, we conducted sensitivity analyses using responses from both the 2011 and 2014 waves as alternative measures.

### 2.4 Covariates

We selected common demographic and socioeconomic factors potentially affecting CVH in older adults as covariates (32). The covariates include sex (male / female), age (as an integer continuous variable), years of schooling (as an integer continuous variable), marital status (married / non-married), income (quantile), race (han / non-han), and living arrangement (living alone / not living alone). In addition, given that healthcare is a service utilized throughout the life course, CAH may be correlated with Access to Healthcare (AH) in different life stages. This introduces a potential for confounding, where healthcare access at subsequent life stages could influence the observed outcomes. Therefore, we incorporated variables representing healthcare access at different life stages into our model. CLHLS has investigated AH of older adults at age 60 (“Could you get adequate medical service when you were sick at around age 60? “) and at baseline (“Could you get adequate medical service when you were sick at present”), we included these two measures as covariates in our analysis.

### 2.5 Statistical analysis

We first employed the GBTM approach to identify CVH score trajectories, which is one of the most commonly used methods for identifying subgroups within longitudinal data. Compared to alternative methods such as the Latent Growth Mixture Model, GBTM requires fewer computational resources, offers simpler fitting, and is more suitable for use with smaller sample sizes (33). In trajectory analysis, model fit is typically evaluated using statistical measures such as the Bayesian Information Criterion (BIC), Average Posterior Probability (APP), and the proportion of observations in each group (34, 35). Based on these criteria, model selection followed the guidelines: (1) choosing the optimal number of trajectories based on the minimum BIC; (2) computing posterior probabilities for each participant and assigning them to the trajectory group with the highest probability, where an APP exceeding 80% indicates an acceptable model fit; and (3) ensuring that the smallest trajectory group accounted for at least 5% of the total sample.

Kruskal-Wallis and Chi-square test were used to examine the differences in covariates among different trajectory samples. A multi-logistic regression model was used to examine the association between CAH and CVH score trajectories, with all covariates adjusted.

Some studies indicated that most research dealt inadequately with the fact that the association between adult health and early life experience was confounded by persisting social and economic disadvantage (36). To further eliminate potential confounding and sample selection bias, and to enhance the robustness of the conclusions while estimating the causal treatment effects of CAH on CVH, we applied the counterfactual causal framework by entropy balancing to weight the covariates (37). Entropy balancing, an alternative matching technique, adjusts the weights of the control group data to match the covariate distribution of the treatment group. Normally, propensity score matching (PSM) is a more popular method for estimating causal treatment effects (38). However, when the sample size in the treatment group is small, PSM can result in substantial sample loss, which may significantly impact the final conclusions (39). Entropy method directly establishes covariate balance in the weighting function, ensuring no sample loss. Compared to other techniques like propensity score matching, entropy balancing performs better in terms of bias reduction and mean squared error (40). We used the EBALANCE package in Stata to directly balance the covariates (41).

## 3 Results

### 3.1 Trajectories of CVH score

**Figure 2:**
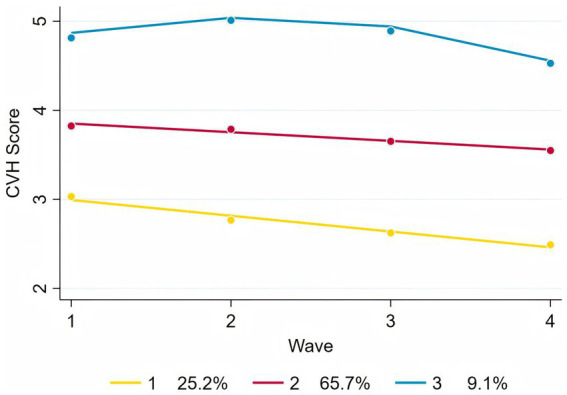
Trajectories of CVH score.

Following model selection based on goodness-of-fit criteria (Tables 2, 3), we divided the sample into three distinct trajectories with significant differences (Figure 2). The Group 1 (Low-rapid decline, approximately 25.2%) had an initial CVH score (intercept) of 3.17, and a linear slope of −0.18, indicating a continuous decline in CVH over time. This suggests that individuals in this group experienced a significant deterioration in CVH during the study period. The Group 2 (Moderate-stable, approximately 65.7%) started with a relatively higher score (intercept of 3.95), but declined at a slower rate with a slope of −0.10. This group represents the largest population in the study, where health levels were still acceptable but showed mild risk of decline. The Group 3 (High-stable, approximately 9.1%) had the highest baseline score (intercept of 4.41), with a significant positive linear term (0.66), suggesting an early upward trend, but with a negative quadratic term (−0.15), indicating a later decline. However, their overall health remained the best among the three groups.

**Table 2:** Performance of group-based trajectory model.

Fit statistic	Number of classes				
	2	**3**	4	5	6
BIC	−11417.40	**−11305.81**	−11297.21	−11270.86	−11270.28
AIC	−11389.42	**−11263.84**	−11241.25	−11200.91	−11186.34
loglik	−11379.42	**−11248.84**	−11221.25	−11175.91	−11156.34
Entropy	0.55	**0.68**	0.75	0.74	0.75
Class proportion (%)					
1	53.32	**26.20**	26.17	16.59	0.43
2	46.68	**65.32**	64.28	61.82	22.94
3		**8.48**	1.91	18.49	59.62
4			7.64	2.27	13.93
5				0.84	2.26
6					0.81
APP					
1	0.87	**0.80**	0.81	0.78	0.87
2	0.85	**0.87**	0.88	0.83	0.81
3		**0.83**	0.76	0.80	0.82
4			0.81	0.78	0.78
5				0.93	0.77
6					0.91

BIC, Bayesian Information Criterion; AIC, Akaike Information Criterion; loglik, Log-likelihood; Lower BIC/AIC and higher loglik suggest better model fit. Entropy indicates classification accuracy (0–1); values closer to 1 reflect better separation between classes. Class proportion (%) shows the estimated size of each latent class. APP, Average Posterior Probabilities; higher APP (>0.80) indicates better classification certainty. Although the BIC values were smaller when the number of trajectories was set to 4, 5, or 6, the guidelines (2) and (3) led us to select 3 groups (the bold column) as the optimal choice.

**Table 3:** Performance of the optimal group-based trajectory model.

Trajectory group	Parameter	Est.	SE	z value	*p* value
Group 1	Intercept	3.171	0.066	47.84	<0.001
(*n* = 486, 25.23%)	Linear	−0.178	0.023	−7.70	<0.001
Group 2	Intercept	3.950	0.050	79.78	<0.001
(*n* = 1,354, 65.69%)	Linear	−0.098	0.013	−7.67	<0.001
Group 3	Intercept	4.412	0.246	17.94	<0.001
(*n* = 150, 9.08%)	Linear	0.657	0.218	3.02	0.003
	Quadratic	−0.154	0.043	−3.61	<0.001
Sigma	–	0.880	0.008	109.82	<0.001

Est., Parameter estimate; SE, Standard Error; *z*-value and *p*-value assess statistical significance of model parameters. Trajectories are modeled using polynomial terms (Intercept, linear, quadratic) to reflect change over time. Sigma, Within-class standard deviation of the outcome variable.

### 3.2 Baseline descriptive analysis

Table 4 presents the results of the descriptive statistics. Significant differences were observed across the groups in terms of CAH (*p* < 0.001), with Group 3 exhibited the highest prevalence of CAH among older adults (50.7%), followed by Group 2 (33.4%) and Group 1 (32.9%). Similar significant differences were also observed for AH (Age 60, *p* = 0.011) and AH (Baseline, *p* = 0.035). Significant differences were observed across the groups in terms of other covariates. In terms of sex distribution (*p* = 0.008), Group 1 and Group 2 exhibited a relatively balanced male-to-female ratio, whereas Group 3 had a significantly higher proportion of males (60.0%). The mean ages of the three groups were similar and the median analysis revealed a marginally significant age difference (*p* = 0.057). Significant differences were found in years of education across the three groups (*p* < 0.001), with Group 3 having significantly more years of education compared to the other two groups (5.2 years vs. 2.8 years). Han ethnicity predominated in all three groups (92.2–96.7%), and the differences between groups were not statistically significant (*p* = 0.161). Income levels also showed significant differences across the groups (*p* < 0.001), with Group 3 having a significantly higher proportion of high-income individuals compared to the other two groups. Differences in living arrangements were not statistically significant (*p* = 0.746), whereas marital status exhibited a marginally significant (*p* = 0.057). In conclusion, significant differences in several sociodemographic characteristics were observed across the trajectory groups.

**Table 4:** Characteristics of participants for trajectories.

Variables	Group 1: low-rapid decline	Group 2: moderate-stable	Group 3: high-stable	Total	χ^2^	*p*
	(*n* = 486)	(*n* = 1,354)	(*n* = 150)	(*n* = 1990)		
CAH (n, %)					18.6	<0.001
Not accessible (=0)	326 (67.1)	902 (66.6)	74 (49.3)	1,302 (67.1)		
Accessible (=1)	160 (32.9)	452 (33.4)	76 (50.7)	688 (32.9)		
AH (Age 60; n, %)					9.0	0.011
Not accessible (=0)	85 (17.5)	189 (14.0)	12 (8.0)	286 (14.4)		
Accessible (=1)	401 (82.5)	1,165 (86.0)	138 (92.0)	1704 (85.6)		
AH (Baseline; n, %)					6.7	0.035
Not accessible (=0)	39 (8.0)	90 (6.6)	3 (2.0)	132 (6.6)		
Accessible (=1)	447 (92.0)	1,264 (93.4)	147 (98.0)	1858 (93.4)		
Sex (n, %)					9.5	0.008
Male (=0)	239 (49.2)	634 (46.8)	90 (60.0)	963 (48.4)		
Female (=1)	247 (50.8)	720 (53.2)	60 (40.0)	1,027 (51.6)		
Age (Mean, SD)	74.0 (7.8)	75.0 (8.1)	74.8 (7.6)	74.7 (8.0)	5.7	0.057
Years of schooling (Mean, SD)	2.8 (3.5)	2.8 (3.5)	5.2 (5.0)	3.0 (3.7)	37.5	<0.001
Race (n, %)					3.6	0.161
Non-han (=0)	38 (7.8)	92 (6.8)	5 (3.3)	135 (6.8)		
Han (=1)	448 (92.2)	1,262 (93.2)	145 (96.7)	1855 (93.2)		
Income quantile (n, %)					44.6	<0.001
1	148 (30.5)	386 (28.5)	20 (13.3)	554 (27.8)		
2	151 (31.1)	374 (27.6)	30 (20.0)	555 (27.9)		
3	86 (17.7)	271 (20.0)	35 (23.3)	392 (19.7)		
4	101 (20.8)	323 (23.9)	65 (43.3)	489 (24.6)		
Living arrangement (n, %)					0.6	0.746
Not living along (=0)	409 (84.2)	1,151 (85.0)	130 (86.7)	1,690 (84.9)		
Living along (=1)	77 (15.8)	203 (15.0)	20 (13.3)	300 (15.1)		
Marital status (n, %)					5.7	0.057
Not married (=0)	297 (61.1)	779 (57.5)	100 (66.7)	1,176 (59.1)		
Married (=1)	189 (38.9)	575 (42.5)	50 (33.3)	814 (40.9)		

SD, Standard deviation.

### 3.3 Entropy balancing process

Table 5 presents the results of the entropy balancing of covariates. Before balancing, the treatment group had clear differences in covariates, which could be important sources of confounding. After entropy balancing, the differences between the treatment and control groups in covariates were significantly reduced. This indicates that entropy balancing successfully balanced these covariates, providing a more reliable foundation for subsequent causal inference.

**Table 5:** Entropy balancing result.

Variables	Before balancing						After balancing					
	Treat (CAH = 1)			Control (CAH = 0)			Treat (CAH = 1)			Control (CAH = 0)		
	Mean	Variance	Skewness	Mean	Variance	Skewness	Mean	Variance	Skewness	Mean	Variance	Skewness
AH (Age 60)	0.97	0.03	−5.94	0.79	0.16	−1.46	0.97	0.03	−5.94	0.97	0.03	−5.89
AH (Baseline)	0.96	0.04	−4.65	0.92	0.07	−3.10	0.96	0.04	−4.65	0.96	0.04	−4.65
Sex	0.48	0.25	0.06	0.53	0.25	−0.13	0.48	0.25	0.06	0.48	0.25	0.06
Age	73.89	56.31	0.97	75.12	67.88	0.91	73.89	56.31	0.97	73.89	58.96	0.99
Years of schooling	3.78	16.46	0.95	2.54	11.75	1.45	3.78	16.46	0.95	3.78	17.62	1.09
Race	0.94	0.05	−3.83	0.93	0.07	−3.26	0.94	0.05	−3.83	0.94	0.05	−3.83
Income quantile	2.57	1.23	−0.04	2.32	1.30	0.27	2.57	1.23	−0.04	2.57	1.33	−0.03
Living arrangement	0.14	0.12	2.13	0.16	0.13	1.87	0.14	0.12	2.13	0.14	0.12	2.13
Marital status	0.59	0.24	−0.35	0.59	0.24	−0.38	0.59	0.24	−0.35	0.59	0.24	−0.35

### 3.4 Logistic regression analysis

Table 6 illustrated the impact of CAH on CVH trajectories. Unadjusted, adjusted, and entropy-balanced models were used to examine how CAH influences the occurrence of three distinct CVH score trajectories. In the unadjusted model, compared to Group 3 (the best CVH trajectory group, as the reference group), individuals with CAH were significantly less likely to experience poorer health trajectories (Group 2 and Group 1). Specifically, individuals with CAH had a 51% lower probability of being in Group 2 (OR = 0.49, 95% CI: 0.35–0.69, *p* < 0.01) and a 52% lower probability of being in Group 1 (OR = 0.48, 95% CI: 0.33–0.69, *p* < 0.01). In the adjusted model, after controlling for potential confounders, the protective effect of CAH remained significant. Specifically, individuals with CAH had a 39% lower probability of being in Group 2 (OR = 0.61, 95% CI: 0.43–0.88, *p* < 0.01) and a 36% lower probability of being in Group 1 (OR = 0.64, 95% CI: 0.43–0.95, *p* < 0.05), compared to those without CAH. Entropy-balanced regression analysis further validated these findings, providing stronger causal inferences. After balancing covariates through entropy balancing, the results showed that individuals with CAH were significantly less likely to enter poorer health trajectories (Group 2 and Group 1). In the entropy-balanced model, the probability of being in Group 2 was 39% lower (OR = 0.61, 95% CI: 0.44–0.85, *p* < 0.01), and the probability of being in Group 1 was 37% lower (OR = 0.63, 95% CI: 0.44–0.90, *p* < 0.05), compared to those without CAH. In stark contrast, after controlling for all covariates, neither AH at age 60 nor at baseline showed a statistically significant association with CVH trajectories, underscoring the foundational role of CAH in shaping these long-term CVH outcomes. In Table 7, we also present sensitivity analyses utilizing alternative-year CAH recall measurements, which demonstrate complete consistency in results with our baseline regression estimates.

**Table 6:** Logistic regression results for association between CAH and CVH trajectories.

Variables	Unadjusted		Adjusted		Adjusted & Balanced	
	1 VS 3 OR	2 VS 3OR	1 VS 3 OR	2 VS 3 OR	1 VS 3 OR	2 VS 3 OR
	(95% CI)	(95% CI)	(95% CI)	(95% CI)	(95% CI)	(95% CI)
CAH						
Not accessible	Ref.	Ref.	Ref.	Ref.	Ref.	Ref.
Accessible	0.48***	0.49***	0.64**	0.61***	0.63**	0.61***
	(0.33–0.69)	(0.35–0.69)	(0.43–0.95)	(0.43–0.88)	(0.44–0.90)	(0.44–0.85)
AH (Age 60)						
Not accessible			Ref.	Ref.	Ref.	Ref.
Accessible			0.66	0.90	0.58	0.76
			(0.34–1.31)	(0.47–1.73)	(0.15–2.21)	(0.21–2.71)
AH (Baseline)						
Not accessible			Ref.	Ref.	Ref.	Ref.
Accessible			0.47	0.51	0.42	0.45
			(0.14–1.60)	(0.15–1.67)	(0.11–1.69)	(0.12–1.69)

OR, Odds Ratio; CI, Confidence Interval. ***p < 0.01, **p < 0.05, *p < 0.1.

**Table 7:** Sensitivity analysis.

Variables	Adjusted and entropy balanced	
	1 VS 3 OR (95% CI)	2 VS 3 OR (95% CI)
CAH answer in 2011		
Not accessible	Ref.	Ref.
Accessible	0.48***	0.50***
	(0.34–0.69)	(0.36–0.69)
CAH answer in 2014		
Not accessible	Ref.	Ref.
Accessible	0.58***	0.57***
	(0.41–0.83)	(0.41–0.79)

OR, Odds Ratio; CI, Confidence Interval. ***p < 0.01, **p < 0.05, *p < 0.1.

In conclusion, the results indicate that CAH significantly reduces the probability of entering poorer CVH trajectory groups (Group 2 and Group 1). Whether in the unadjusted, adjusted, or entropy-balanced models, individuals with CAH are less likely to be in the poorer CVH trajectory groups, highlighting the potential public health benefits of improving CAH for long-term CVH.

### 3.5 Subgroup analysis

**Figure 3:**
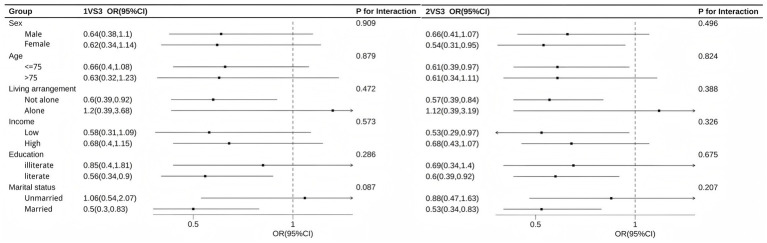
Subgroup logistic regression results for association between CAH and CVH trajectories.

Figure 3 illustrates the results of subgroup analysis. The results indicate that the association was more pronounced among female, age under 75 years old, non-living-alone, low-income, literate, and married individuals. The P for interaction test results show that, with the exception of a marginally significant difference by marital status in the 1VS3 comparison (*p* = 0.087), the differences between subgroups for all other stratifying variables were not statistically significant, suggesting that the association is generally stable across different populations.

## 4 Discussion

This study provides valuable insights into the long-term CVH trajectories of Chinese older adults. We identified three distinct CVH trajectories: Low-rapid decline, Moderate-stable, and High-stable. CAH was associated with better CVH outcomes.

To our knowledge, this is the first study to examine CVH trajectories in Chinese older adults. We found that CVH scores showed a gradual decline over the study period, but exhibited distinct patterns in both baseline levels and rates of decline. These findings support the notion that CVH deteriorates with advancing age (42), and demonstrate that CVH trajectories in older adults differ from those observed in other age groups (10). A meta-analysis has shown that meeting 5–7 (7 in total) CVH metrics provides the greatest protection for health while lowering the risk of CVD; however, maintaining 3–4 metrics also offers a significant protective effect (43). The three trajectories we identified in our study support this conclusion. In another study conducted in northern China, CVH was categorized into Inadequate, Average, and Optimum levels. Its conclusions similarly indicated that a higher CVH score is related to a decrease in CVD incidence, and being in a better CVH category was associated with 47% reduced odds of CVD events (44). The finding also aligns with previous studies suggesting that early-life healthcare access can influence long-term health outcomes (17, 45). Similar studies conducted in western populations have shown that CAH reduces the risk of CVD in later life (46). This study extends long-term pattern research of CVH in China, and highlighting the importance of CAH in the prevention of long-term CVH decline.

CAH may influence CVH in older adults through several interconnected mechanisms. CAH can improve long-term health outcomes by promoting the prevention and treatment of chronic conditions during childhood that increase cardiovascular risk, such as hypertension, diabetes, and obesity (47–49). Studies suggest that children who receive early interventions in managing conditions like high cholesterol, and metabolic disorders are less likely to develop cardiovascular issues in later life (50, 51). On the other hand, lack of CAH can lead to delayed diagnoses, and poorer management of childhood diseases, which could increases the risk of cardiovascular diseases in later life (52, 53). Furthermore, CAH fosters the development of health-promoting behaviors (54), which are crucial for CVH in adulthood (55). Early exposure to healthcare systems helps create better health literacy, which influences lifelong health behaviors and decision-making regarding preventive care (56). These early-life healthcare experiences, therefore, can either mitigate or exacerbate the risks of CVD in older adults, depending on the quality and consistency of CAH.

This study offers actionable implications for China’s healthcare system amid rapid population aging. In the absence of adequate childhood healthcare treatment, older adults not only exhibit lower baseline levels of CAH in later life but also experience accelerated CVH decline. Since the beginning of the 21st century, China has made substantial progress in child health security, representing a significant improvement in both health outcomes and medical coverage compared to older adults born six decades ago (57). However, persistent disparities in childhood healthcare access remain prevalent among disadvantaged populations. A cross-sectional study further highlights pronounced inequalities in child health access across different demographic groups and geographic regions in China (58). A recent study revealed that the substantial rise in incident CVD cases and mortality rates underscores the persistent burden of CVD, which disproportionately affects older adult populations (42). Advanced aging population serves as a significant exacerbating factor that amplifies the public health impact of CVD, with distinct epidemiological implications. With an estimated 418 million individuals aged over 65 by 2035, addressing CVH decline through life-course interventions is imperative. The findings advocate for prioritizing equitable access to childhood healthcare, particularly in low-income regions, to mitigate later-life health disparities. Strengthening primary healthcare infrastructure in underserved areas could ensure early disease prevention and health behavior cultivation, aligning with China’s “Healthy China 2030” goals of reducing CVD burdens (59).

This study has several significant strengths. GBTM model allows for the identification of distinct long-term patterns in CVH, which captures the dynamic nature of CVH over time and provides a deeper understanding of how CVH evolves. The application of advanced statistical methods, including entropy balancing before logistic regression, helps to control for confounding factors and estimate causal treatment effects. This study has several limitations. First, CAH was assessed based on a self-reported survey, which may be subject to recall bias and subjective evaluation. Some studies suggest that in certain scenarios, people perceive their healthcare needs better than healthcare professionals (60). However, data based on self-report may still be affected by recall bias and the judgments of older adults, especially when asking them to recall their childhood healthcare environment from over 60 years ago, which is highly subjective. This is the case even though we attempted to enhance the robustness of our conclusions using repeated measurement data. Furthermore, a comprehensive assessment of CAH involves multiple dimensions and is not merely a “yes” or “no” binary question. Elements such as the quality and efficiency of healthcare services, as well as the severity of illness and individual needs of the older adults, should also be taken into consideration (61). Future research should endeavor to investigate the childhood healthcare conditions of older adults by combining both subjective and objective measures (62). The second limitation of this study is the sample selection. Previous research has shown that the CVH of older adults deteriorates significantly during the last few years of life, with various CVD becoming particularly pronounced (63). This could potentially bias the study’s conclusions. Therefore, we only included older adults who had survived for all four waves. However, a potential issue with this approach is that the included sample may be subject to a selection bias, which could lead to a more conservative estimate of the effect. At the same time, the exclusion of these participants may also weaken the national representativeness of our sample. Future research should consider including a broader sample and further investigate whether the healthcare environment in childhood affects CVH during the end-of-life stage. Finally, while we utilized entropy balancing to control for confounding variables and estimate causal treatment effects, it is important to note that observational studies like this one cannot fully establish causality. Unmeasured or residual confounders may still influence the observed associations between CAH and CVH trajectories. Future research should explore the specific pathways through which CAH influences long-term CVH trajectories, such as its impact on lifestyle behaviors and chronic disease prevention. Additionally, studies that include more diverse populations across different regions of China and longitudinal data are needed to strengthen the generalizability and causal inference of these findings.

## 5 Conclusion

This study highlights the significant association between CAH and CVH trajectories in older adults in China. Our findings suggest that CAH plays a crucial role in CVH outcomes in later life, with those having CAH demonstrating more favorable CVH score trajectories. Given the rapid aging of the Chinese population, these findings highlight the need for public health policies that prioritize CAH as a long-term investment in population health. Policymakers should focus on expanding healthcare access in early childhood to mitigate future healthcare burdens and improve quality of life in older adults.

## Data Availability

Publicly available datasets were analyzed in this study. This data can be found at: https://opendata.pku.edu.cn/dataset.xhtml?persistentId=doi:10.18170/DVN/WBO7LK.
